# Simultaneous Dual Distal Radial Balloon Aortic Valvuloplasty for Larger Aortic Annuli

**DOI:** 10.14797/mdcvj.1147

**Published:** 2022-09-29

**Authors:** Alexandru Achim, Viktor Sasi, Tamás Szűcsborus, Kornél Kákonyi, Zoltan Ruzsa

**Affiliations:** 1 Department of Internal Medicine, Invasive Cardiology Division, University of Szeged, Szeged, Hungary; 2 “Niculae Stancioiu” Heart Institute, University of Medicine and Pharmacy “Iuliu Hatieganu,” Cluj-Napoca, Romania

**Keywords:** distal radial access, snuffbox approach, aortic stenosis, balloon aortic valvuloplasty, vascular complication, bilateral transradial, simultaneous balloons

## Abstract

Dual distal mini-balloon aortic valvuloplasty stabilized an 85-year-old patient with severe aortic stenosis. Puncturing both radial arteries solves the issue of large diameters at the aortic ring, introducing a feasible strategy in selected cases of fragile octogenarian patients with a high hemorrhagic risk. Moving at the anatomical snuffbox offers better postprocedural occlusion rates and better workspace ergonomics during the procedure.

## Background

The predominant access site for balloon aortic valvuloplasty (BAV) is the femoral artery, although the radial artery wall can safely accommodate devices larger than its nominal size regardless of age, body weight, and vessel anatomy. Newer, more flexible balloons that reach a maximum of 22 mm in diameter are now compatible with 6F to 8F sheaths (Table 1); therefore, most cases of BAV can be performed by radial approach, which reduces the vascular complications adjacent to the classical femoral access.^1^ As an effort to further reduce vascular complications (eg, hemostasis time and radial artery occlusion) and offer better intraoperative ergonomics (since the left radial approach can be brought closer to the operator), distal radial access (DRA) BAV showed high technical and clinical success.^2^ For larger aortic annuli, mainly over 24 mm, the efficiency of these balloons is modest in terms of mean invasive gradient reduction, even if their semi-compliant material enables some variation in the diameter above or below the nominal level. Despite the fact that undersizing the valvulotomy is recommended, a 0.8 annulus-to-balloon ratio still cannot be achieved and has a negative impact on the long-term results. To address this constraint and avoid losing the benefits of DRA in BAV, we present a case of BAV performed via bilateral DRA using simultaneous deployment of two balloons (Video 1).

**Table 1:** Low profile 7F- to 8F-compatible valvuloplasty balloons suitable for radial access.


BALLOON NAME	COMPANY	SHEATH SIZE (F)	SHAFT LENGTH (cm)	DIAMETER (mm)	NOMINAL PRESSURE (atm)	RATE BURST PRESSURE (atm)

VACS II	Osypka AG, Germany	7	100	16	2	3

Tishak II	NuMED, Inc., USA	7	100	16	2	2.5

Tishak II	NuMED, Inc., USA	7	100	17	2	2.5

Valver	Balton, Poland	7	110	15	2.5	5

XXL Balloon Catheter*	Boston Scientific, USA	7	75	16	5	5

VACS II	Osypka AG, Germany	8	100	18	1.5	2

VACS II	Osypka AG, Germany	8	100	20	1.5	2

VACS II	Osypka AG, Germany	8	100	22	1.5	2

Tishak II	NuMED, Inc., USA	8	100	18	1.5	2

Tishak II	NuMED, Inc., USA	8	100	20	1.5	2

Tishak II	NuMED, Inc., USA	8	100	22	1.5	2

Z-Med	NuMED, Inc., USA	8	100	12	6	7

Vida PTV	BD Interventional, USA	8	100	18	4	6

Cristal Balloon	BALT Extrusion, France	8	110	18	5	8

Cristal Balloon	BALT Extrusion, France	8	110	20	5	8

Valver	Balton, Poland	8	110	18	2.5	5

Valver	Balton, Poland	8	110	18	2.5	5

Altosa-XL	AndraTec, Germany	8	110	16	6	9

Altosa-XL	AndraTec, Germany	8	110	18	4	8

Altosa-XL	AndraTec, Germany	8	110	20	4	8

XXL Balloon Catheter*	Boston Scientific, USA	8	75	18	5	5


* Peripheral dilatation catheter.

**Video 1:** From a clean puncture to a safe valvulotomy, a step-by-step video on performing dual distal radial mini-balloon aortic valvuloplasty; also at https://youtu.be/IFR9sInuScI.

## Presentation

**Figure 1:**
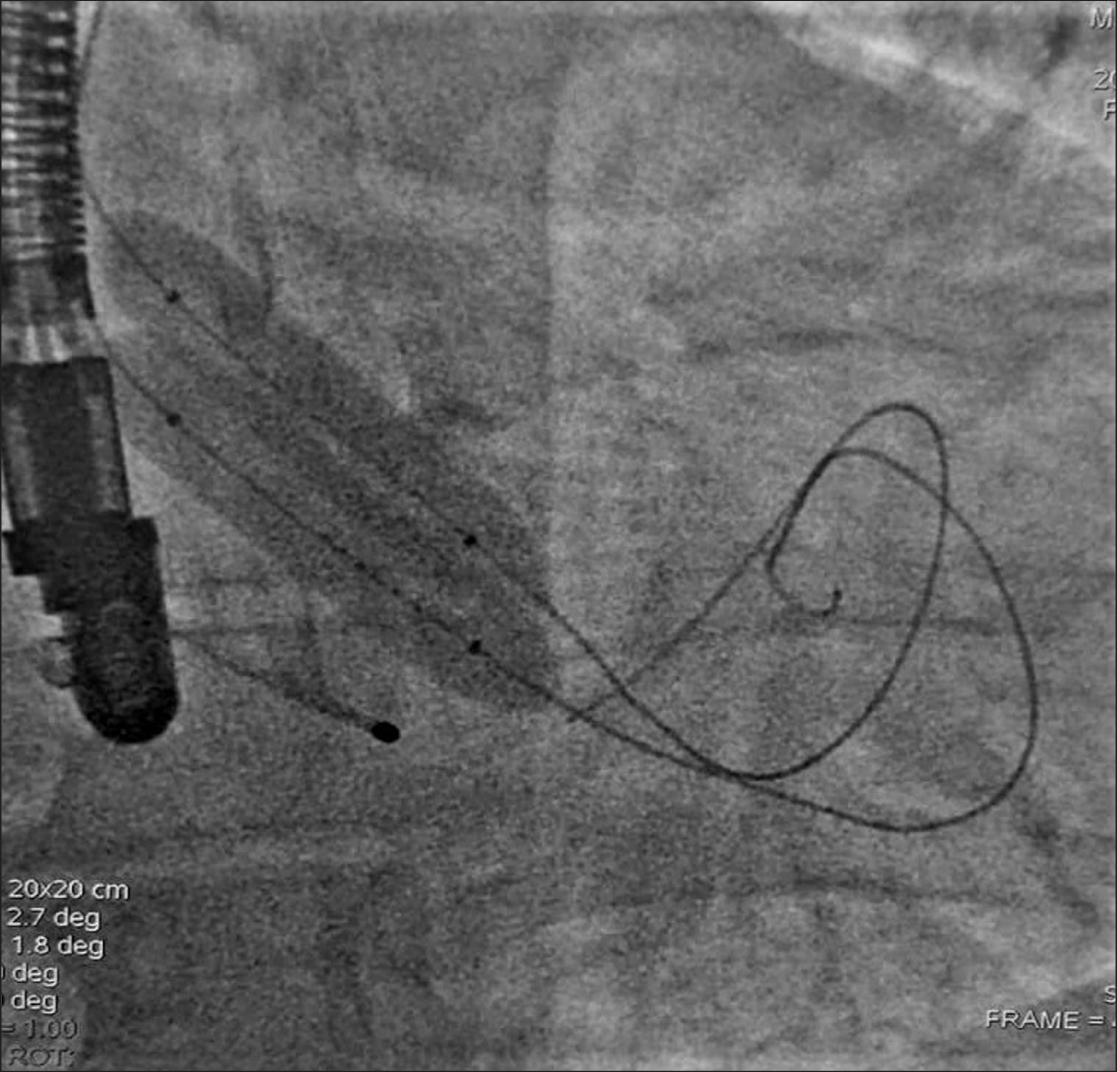
Dual distal radial simultaneous balloon aortic valvuloplasty.

A frail 85-year-old obese male with severe aortic stenosis, paroxysmal atrial flutter, heart failure with reduced ejection fraction (40%), hypertension, type 2 diabetes, stage III/IV chronic kidney disease, and recent gastrointestinal bleeding presented with pulmonary edema. After evaluation, the heart team determined he could not safely undergo transcatheter aortic valve implantation (TAVI) due to his hemodynamic and respiratory instability. Another limitation was that a gastrointestinal endoscopy was deemed necessary before any antiplatelet therapy initiation. The intended strategy was therefore a simultaneous inflation of two 12-mm dedicated balloons (mean annulus diameter of 25.6 mm measured on computed tomography, minimum diameter 24 mm) via bilateral distal radial (6F). Distal “snuffbox” access was chosen over conventional radial access for patient comfort. The patient received 5,000 IU of unfractionated heparin, and no vasodilators were administered at any point of the procedure. After measurements of peak-to-peak transvalvular aortic gradient, an Amplatz Super Stiff™ 0.035” 260-cm guidewire (Boston Scientific, USA) was delivered into the left ventricle over a diagnostic Amplatz left catheter. BAV was performed using two 12- × 40-mm VACS-II balloons (Osypka AG, Rheinfelden, Germany), which were simultaneously inflated under rapid “on-the-wire” left ventricular pacing (Figure 1, Video 1). The invasive mean gradient reduced from 95 mm Hg to 34 mm Hg, and the patient improved significantly. The mild aortic regurgitation remained similar after the valvulotomy. He was discharged after 5 days and received definitive TAVI 1 month later.

## The Rationale

It is important to mention that the balloons are chosen according to the area and not the diameter. For example, a 24-mm aortic annulus has an area of 452 mm^2^, while 2- × 12-mm balloons have an area of only 226 mm^2^. The balloons are somewhat slippery, and a stiffer wire can help stabilize the balloon by pushing on the wire against the left ventricle to anchor the balloon and perform a longer pacing run before inflating it. Usually, at least two inflations are required to achieve a significant gradient drop. Additional force is required for balloon retrieval, so two operators are recommended. In some cases, the use of sheathless guides or long sheaths allows balloon manipulation when delivery and retraction are anticipated to be difficult. The decision to use such long catheters is based on ultrasound examination because small-caliber and diseased arteries are prone to additional spasm and device entrapment. In our case, a single 22-mm balloon would have been another solution, but it requires an 8F sheath, which is difficult to achieve in a radial artery (the average diameter of the artery is about 2.5 mm to 2.7 mm while an 8F sheath is over 3 mm).^3^ The ultrasound can also detect calcification in the vessel wall, which significantly limits the versatility of the artery.^4^ If small diameters are found (under 2.0 mm), dual DRA-BAV can prevent crossover to the femoral approach even in the presence of normal aortic diameters (< 24 mm) but where the radial artery cannot accommodate sheaths of 7F to 8F.^2^ In situations of large sheaths, the Cristal balloons (BALT Extrusion, France) theoretically have an advantage as they may be used for radial access with a one-number-smaller introducer sheath size as per “instructions for use.” If the balloon ruptures or the patient presents severe arterial spasms, the whole system should be retrieved “en bloc,” cautiously. With our distal radial experience, we had no balloon entrapment complications.^2^

The rationale of BAV has resurfaced with the increasing prevalence of elderly frail patients with aortic stenosis and the expansion of the TAVI indication (Class Ia) for most patients over 70 years of age.^5,6^ Radial BAV may be used with high-risk patients who are unable to undergo TAVI or surgical aortic valve replacement to bridge to a more stable clinical condition, allowing for a more careful evaluation and treatment decision.^7^ Second, BAV may have diagnostic value in low-gradient severe aortic stenosis with reduced left ventricular ejection fraction, allowing physicians to determine if the myocardium is capable of recovering once the stenosis-induced afterload is removed.^7^ Another reason would be to bridge to a decision before urgent major surgery in patients with an ambiguous prognosis. However, because of its limited duration and efficiency, BAV exposes patients to periprocedural risks and vascular complications that must be considered. This is reflected in its IIb level of recommendation,^5^ therefore the planning must be made carefully within the heart team.

## Conclusion

Puncturing both radials solves the issue of large diameters at the aortic ring, introducing a feasible stratagem in selected cases of fragile octogenarian patients with a high hemorrhagic risk whose femoral arteries must be retained for early bridge-to-TAVI final therapy. The advantages of puncturing the radial at the anatomical snuffbox are the lower rate of radial artery occlusion (high with larger sheaths) and better ergonomic workspace; the hands are side by side, an important and practical detail when the balloons need to be inflated simultaneously.
